# Clustering of health-related behaviors, health outcomes and demographics in Dutch adolescents: a cross-sectional study

**DOI:** 10.1186/1471-2458-13-1118

**Published:** 2013-12-04

**Authors:** Vincent Busch, Henk F Van Stel, Augustinus JP Schrijvers, Johannes RJ de Leeuw

**Affiliations:** 1 Julius Center for Health Sciences and Primary Care, University Medical Center Utrecht, Universiteitsweg 100, STR. 6.131, Postbus 85500, 3508 GA, Utrecht, The Netherlands

## Abstract

**Background:**

Recent studies show several health-related behaviors to cluster in adolescents. This has important implications for public health. Interrelated behaviors have been shown to be most effectively targeted by multimodal interventions addressing wider-ranging improvements in lifestyle instead of via separate interventions targeting individual behaviors. However, few previous studies have taken into account a broad, multi-disciplinary range of health-related behaviors and connected these behavioral patterns to health-related outcomes. This paper presents an analysis of the clustering of a broad range of health-related behaviors with relevant demographic factors and several health-related outcomes in adolescents.

**Methods:**

Self-report questionnaire data were collected from a sample of 2,690 Dutch high school adolescents. Behavioral patterns were deducted via Principal Components Analysis. Subsequently a Two-Step Cluster Analysis was used to identify groups of adolescents with similar behavioral patterns and health-related outcomes.

**Results:**

Four distinct behavioral patterns describe the analyzed individual behaviors: 1- risk-prone behavior, 2- bully behavior, 3- problematic screen time use, and 4- sedentary behavior. Subsequent cluster analysis identified four clusters of adolescents. Multi-problem behavior was associated with problematic physical and psychosocial health outcomes, as opposed to those exerting relatively few unhealthy behaviors. These associations were relatively independent of demographics such as ethnicity, gender and socio-economic status.

**Conclusions:**

The results show that health-related behaviors tend to cluster, indicating that specific behavioral patterns underlie individual health behaviors. In addition, specific patterns of health-related behaviors were associated with specific health outcomes and demographic factors. In general, unhealthy behavior on account of multiple health-related behaviors was associated with both poor psychosocial and physical health. These findings have significant meaning for future public health programs, which should be more tailored with use of such knowledge on behavioral clustering via e.g. Transfer Learning.

## Background

Health-related behaviors such as smoking, peer bullying, alcohol use and unhealthy nutritional habits contribute significantly to the public health burden of major, contemporary diseases such as diabetes, cardiovascular disease and psychiatric and psychosocial disorders. Many of such behaviors originate during adolescence and frequently lead to impaired adult health [1,2]. Recent studies show that several of such health-related behaviors influence each other in a clustered fashion instead of acting independently on one’s health [3-7]. Such clustering has important implications for research and practice due to the resulting synergistic effects, meaning that particular behaviors share a certain variance, resulting in the fact that changing one behavior affects prevalence of another [8,9]. Certain behaviors increase the likelihood of being involved in other risk behaviors [10], e.g. alcohol users are more likely to partake in smoking use than non-drinkers [9]. Such synergistic effects have been shown to increase disease risk to a level greater than either factor alone [3-5,8,9]. The underlying hypothesis behind this is that on top of the health risks that come from a certain behavior, one’s mindset and decision-making processes are affected by partaking in a certain behavior [9].

This has important implications for preventive interventions, because “if there is covariance between these behaviors, then programs that fail to engage multiple risk behaviors are unlikely to be successful or to generate lasting effects” [11]. When behavior A and B cluster, then intervention on behavior A might affect behavior B, even though that was not directly targeted. Conversely, when behavior B is left out, intervening on behavior A might be less effective than a combined approach. Interventions that simultaneously tackled clustered health behaviors have been shown to be more effective as well as less costly [6,10,12].

Such intervention tailoring requires knowledge on the clustering characteristics of a broad scope of health behaviors. However, most past studies on health behavioral clustering focused on a relative small range of health behaviors. They mostly focused on the clustering of nutrition, smoking and exercise [8,13], while some additionally included alcohol use [11,14], safe sex [9,15] or sedentary time [16-18]. However, few studies thus far included behaviors such as bullying/being bullied and/or screen time use (watching TV, playing videogames, using the internet/PC), while their relevance to adolescent health has become increasingly evident [19-26]. Especially the “compulsive aspect” of screen time use has been overlooked thus far, while this is increasingly shown to affect both adolescents’ physical and psychosocial health [19-22,27,28]. Therefore, a better understanding of the interrelations of a broad, *comprehensive* scope of health behaviors is needed [12]. In addition, despite evidence that several health-related behaviors can negatively affect one’s physical and mental health, thus far studies have generally focused only on the associations of such clusters of health behaviors with physical health (mostly on overweight). The relations with psychosocial factors (e.g. self-efficacy or resilience) are underexposed, while they are often targets of health promoting interventions [29,30]. Also, only few studies have focused on adolescents as the population of interest, while they form such a unique population in which many health-related behavior habits find their origin [1,2,31].

Therefore, this study aimed to identify clustering of a comprehensive number of health-related behaviors in adolescents, and, subsequently, to identify groups of adolescents with similar behavior and health outcomes.

## Methods

### Sample and procedures

Students from different educational levels, ethnic backgrounds and ages of five middle-large Dutch high schools (adolescents, 11–18 years old) received an online questionnaire in September 2012. These schools form a convenience sample with all schools participating out of intrinsic motivation, without being provided with funds or other incentives to participate. All schools were assisted with the questionnaire procedures by the research team. All schools are situated in suburban areas of middle to large cities in the Netherlands, are categorized as in-between rural and urban, and include students from both urban and rural background. Students completed the survey individually during class. Survey procedures were designed to allow students to participate *voluntarily* and *anonymously*. Students were made aware that all data were collected confidentially and stored under a password protected website, only to be assessed by the direct researchers and to be processed anonymously. Students and parents were informed on the nature and format of the survey in timely fashion and were made explicitly clear that participation was voluntary. Approximately 80% of the eligible students completed the survey. Only students with conflicting course schedules (according to their teachers) or those that were ill/absent on the day of the survey did not participate. Since the actual day and timing of the surveys was unannounced, selection bias was prevented in this step. This study has been approved by the Medical Ethics Committee of the University Medical Center Utrecht (UMCU), The Netherlands. METC-protocol number 11–397 / C. For this study the UMCU’s Medical Ethics Committee decided no informed consent procedure was necessary, due to the coded data.

### Measures

Data consisted of self-report data from a sample of Dutch high school adolescents on their relevant demographics, a range of health behaviors and several health outcomes. All outcomes concerned the individual students as the primary sampling unit of the study. The questionnaire was based on the Dutch version of the WHO’s Health Behavior in School-Aged Children (HBSC) questionnaire [32,33]. Table 1 shows the operationalization of all main measures. All behaviors that were included regarded “*Recent behaviors*”, meaning that one indicated to partake in a certain behavior in the month prior to filling out the questionnaire. With regard to bullying this concerned three months prior to filling in the survey. The questions regarding psychosocial problems and self-efficacy were more general, without indicating a certain period of time in which the behaviors had to have taken place.

**Table 1:** The operationalization of the studied health behaviors and health indicators (N = 2,690)

**Variable**	**Item**	**Operationalization**
Cannabis Use [32]	Have you ever used Cannabis (hashish, marihuana or weed)?	0) No, 1) Yes
	Have you recently (in the last 4 weeks) used cannabis (hashish, marihuana or weed)	0) No, 1) Yes
Alcohol use [32]	Have you ever drunk alcohol?	0) No, 1) Yes
	Have you recently drunk alcohol?	0) No, 1) Yes
	How many days a week do you drink alcohol?	Number a day
	How many glasses, cans or bottles of alcohol do you drink a day?	Number a day
Binge drinking [32]	How often in the last 4 weeks have you had more than 4 alcoholic beverages on one occasion?	Number of times, ranging from “Never” to “9 Times or more”.
Smoking [32]	Have you ever smoked a cigarette?	0) No, 1) Yes
	Have you recently smoked a cigarette?	0) No, 1) Yes
	How many cigarettes do you smoke per week?	Categories advancing with 10 pieces per category, ranging from ”0-10” to “70 or more”
Nutrition [32]	How often do you have breakfast per week?	Number of days
	How often do you eat vegetables per week?	Number of days
	How often do you eat fruits per week?	Number of days
Physical exercise [32]	How do you travel to school usually?	0) By bike, 1) Walking, 2) Else
	How long does it take you to walk or bike to school (one-way trip)?	Number of mins
	How many hours of gym class do you have at school each week?	Categories ranging from “0” to “4”.
	Are you a member at a sports club?	0) No, 1) Yes
	How many hours a week do you spend at your sports club?	Number of hours
	How many hours a week do you spend on other sports related activities than previously addressed?	Number of hours
Watching TV [32]	How many days a week do you watch TV?	Number of days
	How many hours a day do you watch TV?	0) Less than 30 mins, 1) 30 mins – 1 hour, 2)1-2 hours, 3) 2–3 hours, 4) >3 hours
Using the internet/PC [32]	How many days per week do you use the internet/PC (not for school purposes)?	Number of days
	How many hours a day do you use the internet/PC (not for school purposes)	0) Less than 30 mins, 1) 30 mins – 1 hour, 2)1-2 hours, 3) 2–3 hours, 4) >3 hours
Videogame playing [32]	How many days a week do you play videogames on a game console?	Number of days
	How many hours a day do you play videogames on a game console?	0) Less than 30 mins, 1) 30 mins – 1 hour, 2)1-2 hours, 3) 2–3 hours, 4) >3 hours
Compulsive internet use [22]	Compulsive Internet Use Scale (CIUS) E.g. Do you rather spend time on the internet than spending time with others? (total: 14 items)	0) Never, 1) Rarely, 2) Sometimes, 3) Often, 4) Very often
Compulsive videogame playing [21]	Videogame Addiction Test (VAT). E.g. Do you rather spend time playing videogames than spending time with others? (total: 14 items)	0) Never, 1) Rarely, 2) Sometimes, 3) Often, 4) Very often
Body Mass Index [32]	How tall are you (no shoes)?	Number of cm
	What do you weigh?	Number of kg
Psychosocial problems [38]	Strengths and Difficulties Questionnaire (SDQ) E.g. I am easily distracted, I find it difficult to focus (total: 20 items)	0) Not true, 1) A little true, 2) Very true
General Self-Efficacy [43]	General Self-Efficacy (GSE) Survey. E.g. If you are going to do something, are you afraid you will fail? (total: 11 items)	0) Never, 1) Sometimes, 2) Often, 3) Very often

The included health-related behaviors were alcohol use, drug use, smoking, physical exercise, nutrition, sexual behavior, screen time (watching television, (online) gaming and internet use) and peer bullying. Most items were surveyed in similar fashion to those of the Dutch HBSC questionnaire; these are summarized in Table 1. The items that differed from the HBSC questionnaire format are discussed in more detail below.

### Bullying

The measurement of bully behavior was based on the Olweus Bully Score and the Olweus Bully Victim Score [37]. These scores distinguish bullies and bullied children from non-bullies and non-bullied children with a cut-off of “2 to 3 times a month”. These measures’ validity and reliability were demonstrated in previous research, stating that these scores allow for prevalence estimates of bullying and being bullied to be obtained conveniently, that they have a reasonably well-defined meaning and that they are easily and unambiguously understood by users and researchers [34].

### Physical exercise and nutrition

Healthy physical exercise was defined as at least one hour of moderately intensive physical exercise every day, where at least twice a week the activity is aimed at improving or maintaining physical fitness. Healthy eating habits were defined as a composite score of having breakfast, eating fruits and vegetables, all at least five times per week. Both are commonly used measures in The Netherlands and the Dutch HBSC [32].

### Screen time: watching television, internet use and videogame playing

Internet use was defined as use of the computer/internet for non-school-related purposes. Videogame playing was defined as (online) gaming on a game console. Spending more than two hours/day on a screen time behavior was defined as “*excessive*” use [22]. The *compulsiveness* of someone’s screen time behavior was measured by the Compulsive Internet Use Scale (CIUS) for compulsive internet use [22] and by the Videogame Addiction Test (VAT) for compulsive videogame playing [21]. The CIUS and VAT both represent a measurement of the core elements of compulsive or addictive behavior that are applicable to Internet use (e.g. loss of control, withdrawal symptoms, coping) [22]. These measures focus particularly on the compulsive and impulse control elements of Internet use and video game playing. To illustrate, issues such as *whether one finds it difficult to stop using the Internet/playing video games*, *whether one rushes through homework to get to using the Internet/video games* or *whether others say one should spend less time on the Internet/playing video games* are questioned by both surveys [21,22]. Both the VAT and CIUS consist of 14 questions with a five-point Likert scale, used to evaluate compulsive behavior, respectively for compulsive videogame playing (CVP) and compulsive internet use (CIU). A mean score higher than 3.0 points indicates compulsive behavior.

### Health outcomes

Three health-related indicators were measured. Firstly, psychosocial problems, which were measured by use of the Strengths and Difficulties Questionnaire (SDQ). This validated questionnaire measures emotional problems, conduct problems, hyperactivity, peer problems, and pro-social behavior, each composed of 5 items scored on a 3-point Likert-scale (0 = “not true”, 1 = “somewhat true” or 2 = “certainly true”). Together, except for the pro-social score, they add up to a total SDQ-score of maximum 40 points [38,39]. A score of 15 of higher is defined as “(potentially) problematic”. The self-report SDQ’s reliability and validity to measure the described psychosocial problem behaviors were recently demonstrated in a comparable sample of Dutch youth [40]. Van Widenfelt, Goedman, Treffers and Goodman later also stated that both the parent and self-report version of the SDQ are acceptable in terms of internal consistency, inter-informant correlations when compared to the “standards”, i.e. the Child Behavior Checklist (CBCL) and the Youth Self Report survey (YSR) [41].

Secondly, “being overweight” was used to indicate unhealthy weight, based on the BMI corrected for age and gender, with appropriate cut-offs in adolescents [42]. Thirdly, due to the importance of *self-esteem*, *social anxiety* and *assertiveness* in adolescent development and psychosocial functioning, a composite measure of these concepts was integrated, which in the literature is referred to as “general self-efficacy” (GSE) [35]. Schwarzer’s conceptual definition of GSE is applied here and that refers to the concept of how one describes his/her beliefs in their capabilities to practice control over challenging demands and regarding their functioning across these domains [43]. The operationalization of GSE measurement that was used was designed for use in Dutch adolescents [43] and based on Rosenberg’s Self Esteem Scale [44] and Schwarzer’s Generalized Self-Efficacy Scale [43]. The GSE questionnaire consisted of 11 questions with a 4-point Likert Scale, a higher score being indicative of more problems. The appropriate cut-off score of higher than 2.50 was used to indicate a problematic score [43].

### Statistical analyses

All statistical analyses were performed with SPSS v20. First, Principal Component Analysis (PCA) was used to identify underlying behavioral patterns from the described health behaviors. Varimax rotation was used. Using the Varimax rotation method minimizes the number of variables that have high loadings on each factor and, as such, simplifies the interpretation of the factors. The extraction of factors in the analysis was based on the Scree Test, a factor loading of at least 0.30 after rotation based on sample size and number of tested variables [36,45] and conceptual meaningfulness [45]. In a follow-up analysis the number of factors to extract was assessed by parallel analysis [46], which compares Eigenvalues of factors from real data with factors from random data. Furthermore, two criteria were tested: the Kaiser-Meyer-Olkin Measure of Adequacy (KMO), a measure of sampling adequacy (threshold: KMO >0.60) and Bartlett’s test of sphericity, which is used to test the null hypothesis that the variables in the population correlation matrix are uncorrelated (threshold: p < 0.05). This PCA produced standardized “component scores” via regression techniques.

Subsequently, a Two Step Cluster Analysis (TCA) was used to identify groups of adolescents with similar behavior and health outcomes [36]. The behavioral patterns (i.e. the component scores derived from the PCA) were used as input variables in the TCA, together with the socio-demographics age, gender, school level, ethnicity, socio-economic status and health outcomes (being overweight, GSE problems and psychosocial problems) [36,45]. A Two-Step Cluster Analysis is used here, due to the mixture of categorical and continuous variables. As stated by Norušis, other cluster analysis approaches will not suffice, since they rely on either continuous or categorical data (hierarchical clustering) or on a preset number of clusters to be distilled (K-means cluster analysis), whereas the TCA can perform an exploratory cluster analysis using a combination of different types of variables [47].

## Results

A total of 2,690 adolescents (response rate 79.8%) aged 11–18 years completed the survey. Students’ characteristics are listed in Table 2. Approximately 45% were boys and the students’ average age was 14 years. Approximately 73% of the 2690 students were native Dutch students; the rest of the students were mostly part of the major ethnic sub-populations of people originating from Morocco, Surinam, Turkey and the former Dutch Antilles.

**Table 2:** Student Characteristics (N = 2,690)

	**N (%)**
Age in years	
11-12	648 (24.1)
13-14	1300 (48.4)
15-16	639 (23.8)
17-18	102 (3.8)
Mean age in years	13.68 (SD 1.45)
Socio-economic status (FAS^a^ score)	7.13 (SD 1.49)
Low (0–2) – Medium (3–5)	393 (14.6)
High (6–9)	2297 (85.4)
Watching TV (>14 h/week)	549 (21.0)
Internet use (>14/week)	730 (27.1)
Videogame playing (>14/week)	186 (6.9)
Compulsive Internet Use, CIU^b^	94 (3.5)
Compulsive Videogame Playing, CVP^c^	108 (4.0)
Being bullied	124 (4.7)
Bullying	77 (2.9)
Alcohol user	702 (26.1)
Binge drinker	477 (19.6)
Marihuana user	170 (6.3)
Smoker	231 (8.6)
Sufficing to Dutch Norm Healthy Physical Exercise^d^	1974 (73.6)
Sufficing to Dutch Norm Healthy Nutrition^e^	1186 (44.1)

^a^:FAS = Family Affluence Scale; ^b^:CIU = Compulsive Internet Use Scale Score >3.0 (range 0–4); ^c^:CVP = Videogame Addiction Test Score >3.0 (range 0–4); ^d^:Dutch Norm Healthy Physical Exercise: at least one hour of moderately intensive physical exercise every day, where at least twice a week the activity is aimed at improving or maintaining physical fitness; ^e^*:*Dutch Norm Healthy Nutrition: at least having breakfast, eating fruits and vegetables 5 times per week.

### Principal component analysis

The sample was considered suitable for factor analysis [36,45], as both the KMO measure (0.69) and Bartlett test of sphericity (p <0.001) exceeded the pre-set threshold. Results of the Principal Component Analysis (PCA) indicated that several separate distinct *behavioral patterns* (the “components” deduced from the PCA) underlie the individual behaviors. From the Scree Plot and further analysis four different behavioral patterns were deduced [36,45]. Selecting more or less than four factors was not in accordance with both the Scree Plot and Parallel Analysis [46], and left a non-interpretable factor solution. The four factors together explained approximately 55% of the total variance of the fourteen analyzed items. Further details of the PCA (item patterns, factor loadings and explained variance) are presented in Table 3. Only the items with a factor loading of at least 0.30 are presented in Table 3. From the PCA four main components were distilled, mainly indicating:

1 Risk-prone behavior (smoking, drug use, alcohol use and sexual activity).

2 Bully behavior (bullying & being bullied and compulsive Internet use).

3 Problematic screen time use (weekly time and compulsiveness of playing videogames & using the Internet).

4 Sedentary behavior (weekly time watching TV and using the Internet), unhealthy nutrition & insufficient physical exercise.

**Table 3:** Factor structure of health behaviors in the study sample (only loadings of above 0.30 presented)

**Behavioral components**	**Factor 1: Risk-prone behavior**	**Factor 2: Bully behavior**	**Factor 3: Problematic screen time use**	**Factor 4: Sedentary behavior**
Smoking	.66			
Cannabis use	.73			
Binge drinking	.78			
Quantity of alcohol use	.80			
Having had intercourse	.65			
Healthy nutrition				-.50
Healthy physical exercise				-.65
Excessively watching TV			.32	.57
Excessive PC/internet use			.32	.65
Excessively playing videogames			.80	
Compulsive PC/internet use		.32	.54	
Compulsive videogame playing			.85	
Being a bully victim		.82		
Being a bully		.86		
Eigenvalue	2.938	2.252	1.423	1.091
Variance explained %	19.132	13.725	11.216	10.959
Cumulative variance explained %	19.132	32.857	44.073	55.032

KMO measure of sampling adequacy = .69 (based on Kaiser’s criteria: KMO ≥ .60 means that the result of the factor analysis is acceptable). Bartlett’s test of sphericity: χ2 = 6853.250 (df = 91; p < .000).

The first factor consisted of the high-risk behaviors alcohol use, drug use, and smoking, and was thus termed risk-prone behavior. The second factor consisted of bullying, being bullied and compulsive PC/Internet use, termed the bully behavior factor. Thirdly, the different aspects of screen time use (i.e. its compulsive component and its excessive use component) formed a separate factor. The fourth factor consisted of the components low physical activity, poor nutrition habits combined with excessively watching TV and using the PC/Internet. This particular aspect of screen time use was related to poor physical exercise and nutrition patterns, whereas the compulsiveness of screen time use had no correlation with those behaviors. This fourth factor was thus termed the sedentary behavior factor. Due to theoretical considerations, and because both loaded above 0.30 in the PCA, compulsive PC/Internet use was included in both factor 2 as well as in factor 3 and excessive PC/Internet use and excessively watching TV were also both included in two different factors (further elaboration upon these choices is presented in the Discussion).

### Two-step cluster analysis

Four clusters were deducted from the Two-step Cluster Analysis (TCA), details are presented in Table 4. Cluster 1 presented the healthiest cluster with the most positive scores in terms of both health behaviors as well as health outcomes. These students were characterized by an average score with regard to sedentary behavior and a low score (i.e. healthy/positive score) on the other three behavioral pattern component scores. This “healthy cluster” was characterized by an average socio-economic status, a mix of different school levels, being of a native Dutch ethnicity and being girls.

**Table 4:** Clusters of health behaviors, health outcomes and demographics, formed by Two Step Cluster Analysis (N = 2,690)

	**Cluster 1**	**Cluster 2**	**Cluster 3**	**Cluster 4**
	35.6%	26.4%	19.8%	18.3%
**Demographics characteristics**				
Gender	Girl (100%)	Boys (100%)	Mixed (58% Girls)	Mixed (58% Girls)
Age	13.6 years	13.7 years	14.0 years	13.6 years
SES (FAS score)^1^	Normal to High (7.27)	High (7.50)	Normal (6.98)	Low (6.38)
School level	Average / Mixed	Average / Mixed	Average / Mixed	Average / Mixed
Ethnicity	Native Dutch (100%)	Native Dutch (100%)	Mixed (81% Native Dutch)	Non- native Dutch (100%)
**Health behaviors**^2,3^				
Factor 1: Risk-prone behavior	Low (−0.16)	Normal (0.09)	High (0.48)	Low (−0.24)
Factor 2: Bully behavior	Low (−0.12)	Low (−0.20)	High (0.60)	Low (−0.19)
Factor 3: Problematic screen time use	Low (−0.39)	High (0.26)	High (0.29)	Normal (0.02)
Factor 4: Sedentary behavior	Normal (−0.06)	Low (−0.20)	High (0.18)	High (0.27)
**Health outcomes**				
Weight status	Normal BMI	Normal BMI	High BMI	Normal to High BMI
Self-efficacy problems	Normal GSE (100%)	Normal GSE (100%)	Problematic GSE (32%)	Normal GSE (100%)
Psychosocial problems	Normal SDQ (100%)	Normal SDQ (100%)	Problematic SDQ (71%)	Normal SDQ (100%)

^1^Indications of low, medium and high are indicative of a relatively low, medium or high socioeconomic status in this particular sample of adolescents.

^2^A low score on a lifestyle factor score indicates less exertion of such behavior.

^3^All Factors are standardized regression scores with a mean of 0 and a standard deviation of 1.

Cluster 2 and 4 were also relatively healthy behaving students but differed in certain aspects from cluster 1. First, cluster 2 had a slightly higher socio-economic status than cluster 1 and consisted only of boys. Also, they presented unhealthier behavior than students of cluster 1 with regard to problematic screen time use (factor 3) and risk-prone behavior (factor 1, although they did not score *above average* on this factor). Cluster 2 also showed healthier behavior with regard to sedentary behavior (factor 4) compared to cluster 1. Furthermore, similar to cluster 1, cluster 2 was also characterized by positive scores on all three health outcomes.

Cluster 4 was similar to the other two “healthy” clusters in terms of scoring positive health outcomes and relatively healthy behavior in terms of risk-prone behavior (factor 1) and bully behavior (factor 2) as well as scoring average on sedentary behavior (factor 4). Typical for cluster 4 was that those students were of a non-Dutch ethnicity, had a low socio-economic status, and consisted of a mix of boys and girls.

Finally, cluster 3 differed from all other clusters. Cluster 3 contained the unhealthiest scores on all four behavioral patterns, as well as the unhealthiest outcomes, namely a high BMI, a problematic SDQ and GSE score. This cluster comprised of students from all ethnicities, an average socio-economic status and school level and consisted of both boys and girls.

## Discussion

This study aimed to identify clustering of a comprehensive number of health-related behaviors in adolescents, and to identify groups of adolescents with similar behavior and health outcomes. Four distinct behavioral patterns were found, namely 1) *risk-prone behavior*, consisting of high/unhealthy scores with regard to smoking, alcohol use, drug use and being sexually active, 2) *bully behavior*, consisting of significant factor loadings from the variables of bullying, being bullied and compulsive internet use, 3) *problematic screen time behavior*, meaning a high/unhealthy score regarding compulsive and excessive screen time use, and 4) *sedentary behavior*, i.e. excessive screen time use combined with poor physical exercise and nutritional habits.

After integrating these behavioral patterns together with several demographic factors and health-related outcomes, four clusters of adolescents were distinguished. One cluster was dominantly the healthy cluster, in which all behavioral pattern scores were all relatively most healthy as well as their situation on account of the health-related outcomes. These students were native Dutch girls from a mix of different socio-economic statuses and school levels. Two other clusters (cluster 2 and 4) differed only slightly from the healthy cluster. Cluster 2 included native Dutch boys, with unhealthy scores on the problematic screen time behavior factor, and average instead of low scores on the risk-prone behavior factor. They also showed positive health-related outcomes. Cluster 4 consisted only of adolescents of non-Dutch ethnicity, mostly with a low socio-economic status, and consisting of a mix of boys and girls. The only behavior pattern in which they scored poorly (i.e. unhealthy) was sedentary behavior (factor 4). Being overweight was also a characteristic of this cluster. Cluster 3 showed strong clustering of both negative health-related outcomes and unhealthy scores on all behavioral patterns, independent of demographic factors.

### Behavioral factors

#### Smoking, alcohol use, drug use and sex

The first deduced distinct behavioral pattern that was the *risk-prone behavior* factor. The individual behaviors that made up this factor (or pattern) were marihuana use, smoking, alcohol use and sexual activity. Of all studied behaviors these four all seem to present a norm-deviating behavior and therefore the factor was named risk-prone behavior. Their correlation is in accordance with previous research, which has mostly focused on these behaviors out of all those that were studied in the current research. Van Nieuwenhuizen et al. for example also showed a strong correlation of substance use related behaviors and sexual behavior with factor analysis techniques, yet due to the different scope of included behaviors these results were only partly comparable to those of the current study [12]. No major differences in behavioral patterns from the study of Van Nieuwenhuizen or other previous studies were found [9,12-15,49].

### Bully behavior

Secondly, being bullied and being a perpetrator of bullying formed a behavioral component in the PCA. Compulsive Internet use also loaded significantly on this factor. Despite the fact that this behavior loaded stronger on another factor (namely on factor 3), it was included in this bully behavior factor also, due to theoretical considerations, since previous research also reported on the relationship between the internet use of adolescents and their bully behavior [50]. Compulsive screen time use, when being bullied, could possibly indicate a kind of ‘flight behavior’ to a relative anonymous online environment in which one would feel safer. Thus, in this context compulsive screen time use seems to be part of a distinctly different overarching behavior than in factor 3 (discussed below). Therefore, it was included in two different factors, as is common practice in factor analyses when theoretical considerations are taken into account instead of merely looking at statistical considerations [36]. However, it has to be taken into consideration that such a theory is relative speculation due to the few comparable other studies on the topic.

Furthermore, the subsequent TCA confirmed their relationship to General Self-Efficacy and psychosocial problems. Students that scored worst on, among other unhealthy behavioral scores, bullying/being bullied also reported the worst psychosocial and GSE outcomes.

### Excessive versus compulsive screen time

In the current study screen time behavior consisted of two aspects, namely excessive and compulsive screen time behaviors. Although these showed to be strongly inter-related (forming a separate behavioral pattern, i.e. factor 3) the associations of excessive and compulsive screen time behaviors to problematic health-related outcomes differed. Excessive screen time was significantly related to being overweight (Cluster 4, Table 3), while compulsive screen time was significantly more prevalent among students that also indicated psychosocial problems, problems with GSE and behaviors such as bullying/being bullied (behavioral factor 2) and risk-prone behaviors (behavioral factor 1) (Table 3). The findings related to excessive screen time behavior were in accordance with previous studies [18,31,48,51]. However, similar clustering studies that integrated the compulsive aspect of these behaviors in adolescents were not retrieved, although previous research has shown that, separately, compulsive and excessive screen time behaviors differ in their relation to outcomes such as psychosocial problems [52], educational outcomes [53] or physical health indicators [23]. Therefore, based on these theoretical considerations, compulsive and excessive screen time behaviors were included in more than one behavioral factor (Table 2).

### Screen time, physical exercise and eating habits

The fourth behavioral pattern was the so-called sedentary behavior factor. Scoring high on this factor meant that students reported more excessive screen time use as well as low levels of physical exercise and unhealthy nutrition habits. Previous studies showed this clustering of nutrition and exercise [11,14,17,18], but relatively few also integrated screen time use. Studies that did, showed relatively similar cluster patterns [16,27,51]. The current study shows that the more hours teens spend on watching television, using the internet and playing videogames, the less time they spend on physical exercise and the poorer they eat. Thus, for public health practice this would mean that solely focusing on e.g. weight reduction via attention for more sports participation and healthier nutrition seems inadequate. The screen time behavior of the children and adolescents of our digital age seems an inescapable phenomenon that has to be integrated in health promotion practices.

### Clustering of health behavior with health outcomes and demographic factors

After deducing the overarching factors/behaviors from the individual behaviors, several noteworthy results were found in the subsequent TCA. Firstly, as predicted by previous studies expected from the literature, being overweight was significantly related to a lower socio-economic status and behavioral factor 4 (sedentary behavior). Poorer scores on this behavioral pattern was one of the few aspects in which clusters 2 and 4 differed from one another, together with non-Dutch ethnicity and the higher correlation to being overweight of cluster 4.

Secondly, the TCA revealed that poor scores on multiple behavioral patterns and poor health outcomes clustered within the same students. This was in accordance with previous comparable studies that showed that more problematic behaviors led to, or were associated with, poorer health outcomes [3,4,8,13,17]. Also, the clustering of compulsive screen time and bully behavior with psychosocial problems and low GSE is in line with other literature [54-56].

Furthermore, boys seem to exert unhealthier behavior than girls in comparable groups when reviewing cluster 1 versus cluster 2, especially with regard to risk-prone behavior and problematic screen time use. This is in line with the theory and findings of the meta-analysis of Byrnes, Miller and Schafer of over 150 studies on the subject that revealed a higher prevalence of risky behavior among males than females [57].

Finally, the clustering of health-related behaviors and –outcomes showed to be independent of demographic factors socio-economic status, gender, ethnicity and school level. This is a finding that would indicate that unhealthy behavior is the main indicator for subsequent poor health-related outcomes and that demographic factors have only a minor influence on this. This is not in accordance with several previous studies that indicated a significant effect of, especially, socio-economic status. It was difficult to assess whether the lack of clustering with socio-economic status in the current study could be specific to the study sample, due to the relatively minor variations in socio-economic status among Dutch adolescents in comparison to those in other countries.

### Strengths and weaknesses

A strength of the current study was that we took into account a broad range of health-related behaviors that were previously not examined simultaneously in such a way. Many previous studies focused on subsets of these behaviors. Also, the use of validated questionnaires is an important strength of this study. Last, the response rate of 79.8% was a respectable one. The fact that certain students did not fill out the questionnaire was mostly attributable to teachers not presenting the questionnaire to their class; the indicated reasons for this were interfering schedules or that it was forgotten by the teacher. Therefore, non-response bias can be assumed to be minimal.

A limitation is the lack of integrating a multilevel structure in the factor and cluster analyses. For more optimal estimates a multilevel approach is preferred, although such approaches are still in their infancy [58]. However, given the strong factor loadings and strong effects that were found, it is highly unlikely that the impact of integrating a multilevel structure would have significantly changed them. Such effects are especially to be expected and relevant when the variables that are dealt with are cross-level latent constructs [58-60]. This means that if one were to measure higher level constructs (e.g. school climate) via individual level measurements, a multilevel approach would be more likely to be beneficial. However, this was not the case in the current study and therefore such approaches were less relevant. A second limitation of the current study is its cross-sectional design, which inhibits establishing any causal relations. Also, this study used a sample of young adolescents from the Netherlands, which is no guarantee for generalization to other countries. Especially the relatively limited variation with respect to socio-economic might limit the possibility for generalization of some results.

## Conclusions

The results show that health-related behaviors tend to cluster, indicating that specific behavioral patterns underlie individual health behaviors. This resulted in the deduction of four distinct behavioral patterns, namely 1) Risk-pronebehavior (alcohol and drug use, smoking and early sexual activity), 2) Bully behavior (bullying, being bullied and compulsive Internet use), 3) Problematic screen time use (excessively watching television and compulsively and excessively playing video games and using the Internet), and 4) Sedentary behavior (low physical exercise, poor nutritional habits and excessively watching television, playing videogames and using the Internet). Subsequently, four clusters of adolescents were identified; multi-problem behavior was associated with problematic physical and psychosocial health outcomes, as opposed to those exerting relatively few unhealthy behaviors. These associations were relatively independent of demographics such as ethnicity, gender and socio-economic status. Overall, this study adds to the current knowledge on how health behaviors cluster within individuals and that certain combinations of behaviors can be used to target high-risk individuals, which were shown to be of significantly higher risk of poorer physical and psychosocial health outcomes.

Additionally, the findings of this study have significant implications for future school-based prevention programs. As Wiefferink et al. suggested, such knowledge on health behavioral clustering can be used to design more effective and feasible school based interventions using Transfer-oriented Learning [61]. Transfer-oriented Learning is said to take place when students apply independently and flexibly what they have learned in a context different to that in which they learned it [61]. This means for example that, if resisting peer pressure would be an important tool to prevent youth from starting smoking, such a skill can also be learnt to be applied in a different context, e.g. when teaching students to resist drug use or to partake in unprotected sex; certain common determinants can be transferred to teachings on different topics. Although a specific behavioral context is still needed to teach knowledge, attitudes and skills, Transfer-oriented Learning does facilitate more feasible school based interventions, because topics can be integrated, which lightens the load on the curriculum. Also, it would increase the outreach that school based interventions could have when multiple behaviors are targeted simultaneously. Given these developments, it is a positive development to see school based interventions move towards a comprehensive, whole school approach that would facilitate a clustered approach to improving health behaviors among children and adolescents [29]. To improve upon current practices in this area, research on the clustering of health behaviors is vital, since it is necessary to identify common determinants across different types of health behaviors. This study therefore adds significantly to the current knowledge.

## Competing interests

The authors declare that they have no competing interests.

## Authors’ contributions

VB and HVS designed the study, performed the data analysis, and wrote the first draft of the article. VB and RDL collected all data. All authors participated in the interpretation of the findings and finalizing the paper. All authors revised and approved the final draft. GS is responsible for study supervision. All authors share equal responsibility for the paper and approve the final manuscript.

## Pre-publication history

The pre-publication history for this paper can be accessed here:

http://www.biomedcentral.com/1471-2458/13/1118/prepub
